# Multimodal Diagnostic Workup of Primary Pericardial Mesothelioma: A Case Report

**DOI:** 10.3389/fcvm.2021.758988

**Published:** 2021-11-19

**Authors:** Jiahui Liu, Zhi Wang, Ying Yang, Yan Xiong, Wei Wang, Jianxing Qiu, Kai Zhao, Bo Zheng

**Affiliations:** ^1^Department of Cardiology, Institute of Cardiovascular Disease, Peking University First Hospital, Beijing, China; ^2^Department of Pathology, Peking University First Hospital, Beijing, China; ^3^Department of Radiology, Peking University First Hospital, Beijing, China

**Keywords:** primary pericardial mesothelioma, contrast-enhanced ultrasonography, echocardiography, case report, magnetic resonance imaging, computed tomography

## Abstract

**Background:** Primary pericardial mesothelioma is an extremely rare tumor, and early identification and accurate diagnosis may improve its clinical outcome.

**Case presentation:** In this study, we reported a case of a 70-year-old woman who presented with dyspnea. Conventional transthoracic echocardiography showed massive pericardial effusion. Contrast-enhanced ultrasonography revealed a hyper-enhancing mass in the pericardium. Further imaging methods, including cardiac MRI and positron emission tomography/computed tomography, showed invasion of the pericardial mass into the adjacent tissues and distant metastases. Pathologic examination of a puncture biopsy specimen finally confirmed the diagnosis of PPM.

**Conclusion:** Pericardial masses are difficult to detect when a large amount of pericardial effusion is present and the mass is small. The combination of multiple modalities plays a meaningful role in identifying PPM.

## Introduction

Primary pericardial mesothelioma is an extremely rare but highly aggressive primary pericardial malignant tumor (1). The onset of symptoms is usually insidious. In most cases, the tumor is diagnosed at a late stage and is not possible to completely remove. Considering the poor prognosis, early detection of the tumor is clinically important. We reported this case to highlight the difficulty in diagnosing primary pericardial mesothelioma (PPM) and the importance of a multimodal diagnostic work-up.

## Case Report

**Figure 1:**
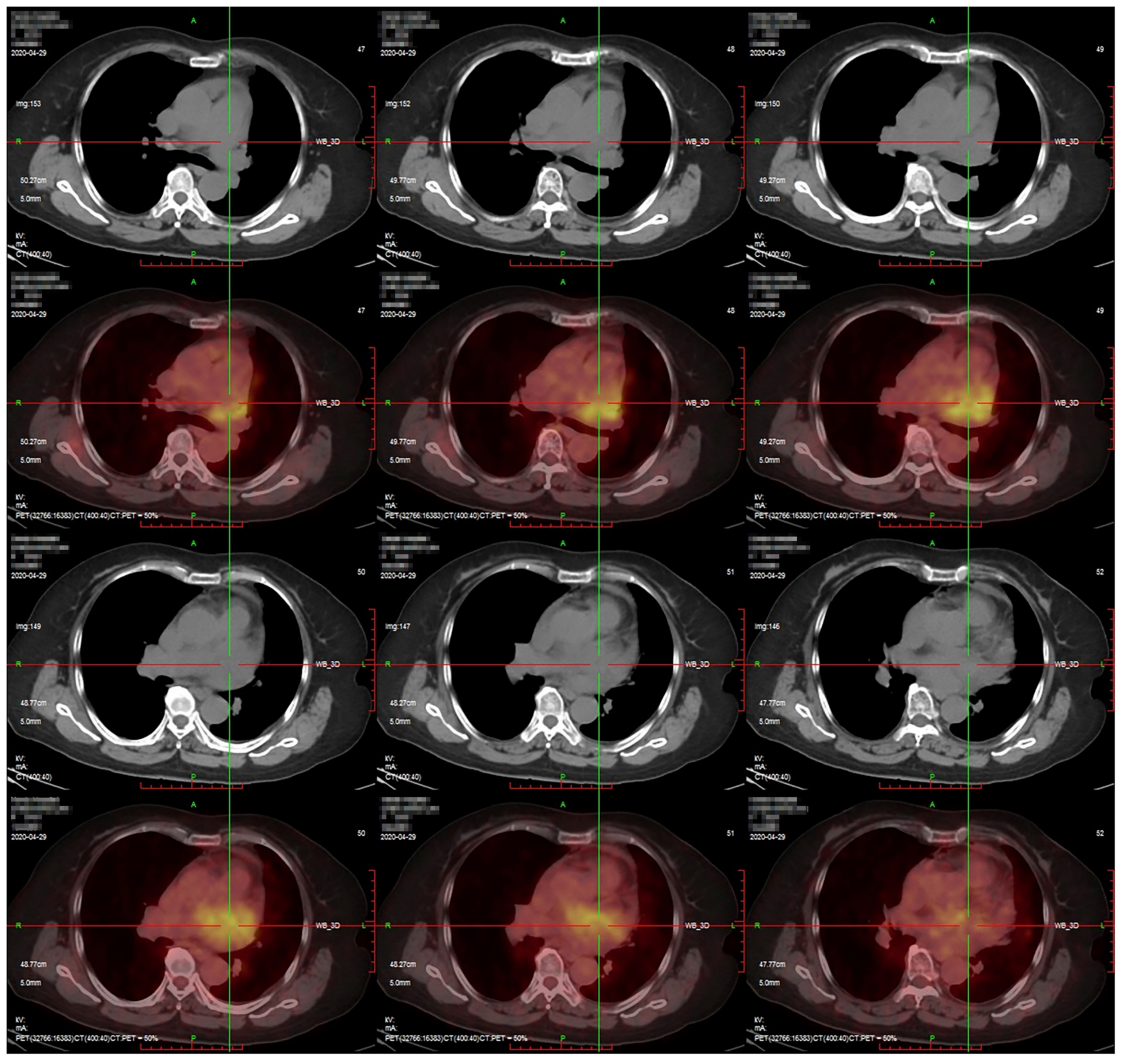
Positron emission tomography/computed tomography findings. Intense fluorodeoxyglucose uptake was observed in the pericardium with a maximum standardized uptake value of 9.6.

In July 2020, a 70-year-old woman presented with progressive dyspnea and was admitted to a local hospital. The patient had no medical history. Blood tests and electrocardiography showed no abnormalities. Transthoracic echocardiography (TTE) showed massive pericardial effusion. Chest enhanced computed tomography (CT) revealed pericardial effusion with no signs of tumors or tuberculosis. The local hospital performed 18F-fluorodeoxyglucose (FDG) positron emission tomography (PET), which showed intense FDG uptake in the pericardium (maximum standardized uptake value: 9.6), the area surrounding the left atrium, and mediastinum (Figure 1). The corresponding CT images demonstrated a mass in the pericardium (1.7 × 1.2 cm) combined with pericardial effusion and scattered soft tissue masses in the area surrounding the left atrium and mediastinum. No distant tumor sites or infiltration were detected. A pericardial window was performed via thoracoscopy, and a limited pericardiectomy was carried out. A pericardial biopsy was performed, and a pathological diagnosis of mesothelial cell hyperplasia was made at the local hospital. The patient was discharged without a definitive diagnosis and was prescribed a diuretic to alleviate her symptoms at home.

**Figure 2:**
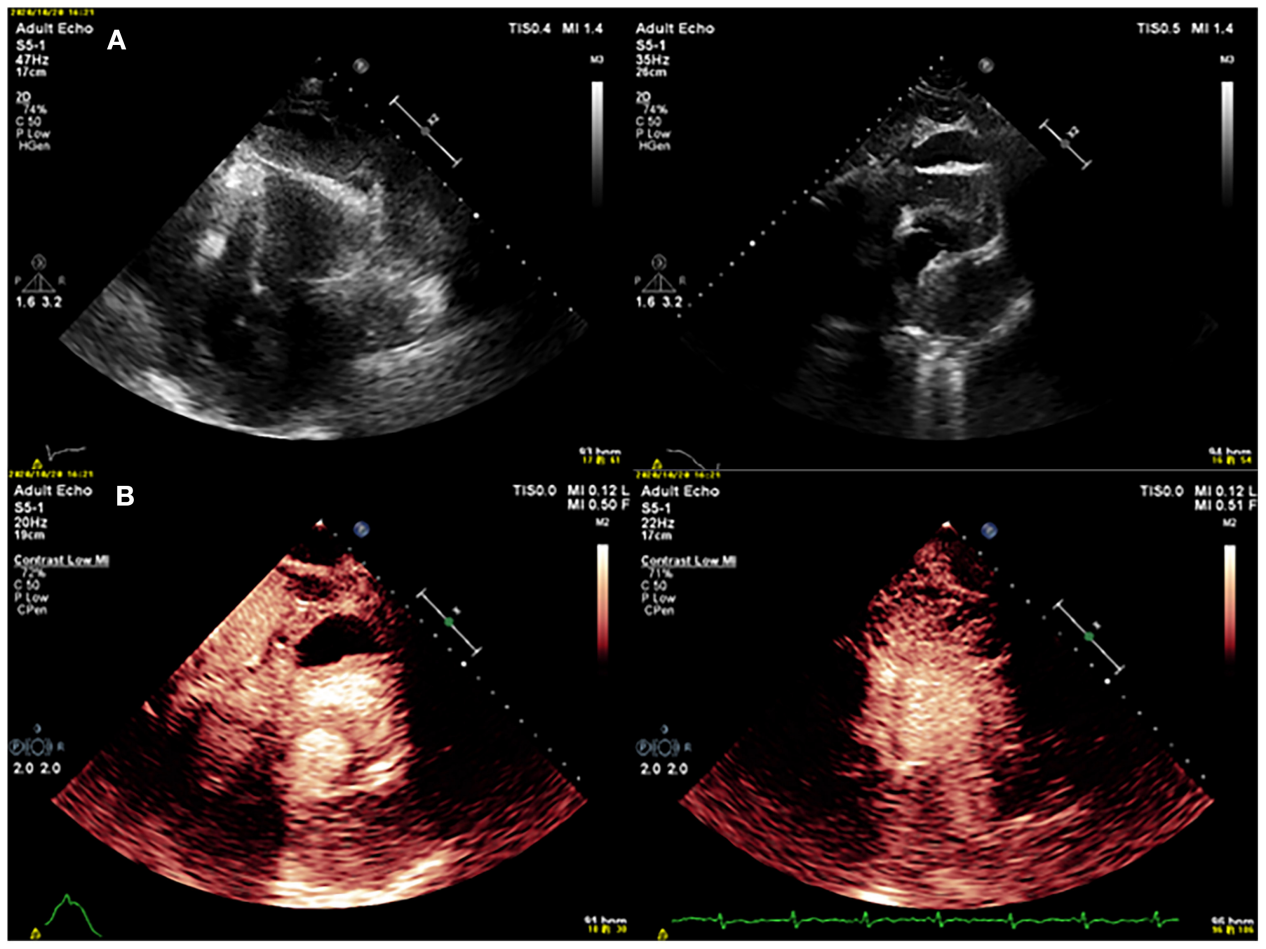
**(A)** Conventional echocardiography showed a mass in the pericardium. **(B)** Contrast echocardiography showed that the mass was hyper-enhancing.

**Figure 3:**
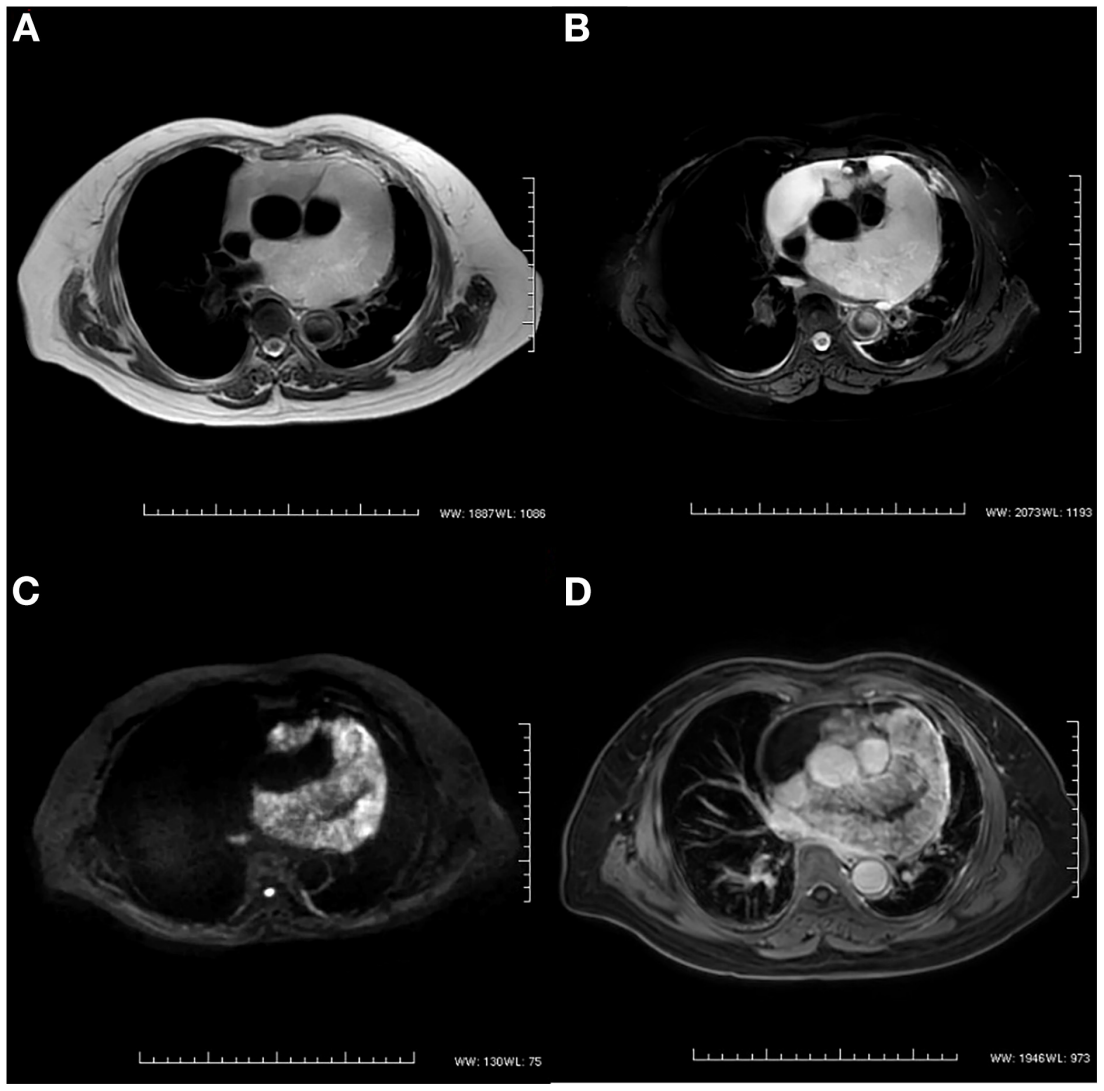
Cardiac magnetic resonance imaging. **(A)** T2-weighted imaging showed an irregular mass in the pericardium. **(B)** The signal intensity of the mass was hyperintense on T2-weighted imaging with fat suppression. **(C)** The diffusion-weighted image was hyperintense. **(D)** The mass showed irregular enhancement with gadolinium injection on T1-weighted imaging.

**Figure 4:**
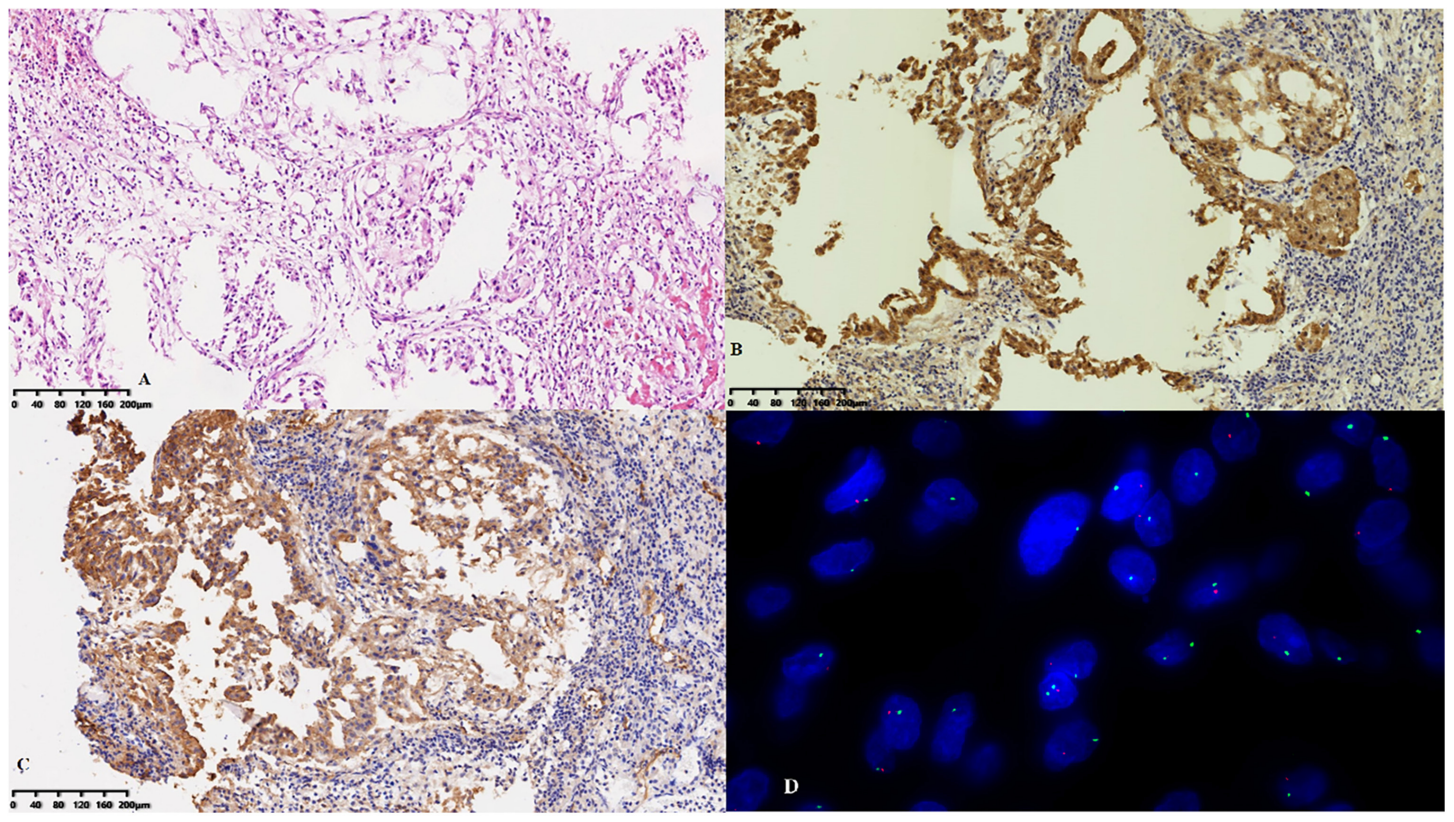
Pathological features. **(A)** Hyperplastic epithelioid cells were arranged in sheets on the surface of the pericardium with an inflammatory background. The cells were positive for **(B)** calretinin and **(C)** D2-40 by immunohistochemistry. **(D)** CDKN2A (p16) deletion was detected by fluorescence *in situ* hybridization.

After 3 months, the patient was referred to our hospital because of worsening shortness of breath and increasing volume of the pericardial effusion. On physical examination, her respiration rate was 25 breaths/min, her pulse rate was 90 beats/min, and her blood pressure was 105/70 mmHg. Reduced breath sounds were heard in both lungs. TTE revealed a homogeneous isoechoic pericardial mass (75 × 30 mm) with massive pericardial effusion (Figure 2A). Neither ventricle was enlarged, and the left ventricular ejection fraction was estimated at 70%. The cardiac valves were morphologically and functionally normal. Contrast-enhanced ultrasonography (CEUS) was then performed with ultrasound contrast agent Sono Vue (Bracco, Italy) intravenous injection. Compared with the adjacent myocardium, the mass was hyper-enhanced (Figure 2B). Further cardiac MRI (CMRI) (Figure 3) showed a pericardial mass attached to the wall of the left atrium with invasion to the pericardium and vessels. The tumor showed irregular enhancement after gadolinium injection. The pericardial biopsy showed that the cells were positive for mesothelial cell markers (calretinin and D2-40) by immunohistochemistry (IHC), and CDKN2A (p16) deletion was detected by fluorescence *in situ* hybridization (FISH) (Figure 4). Finally, the patient was diagnosed with epithelioid mesothelioma of the pericardium. Given the invasion of adjacent tissues and metastasis, the cancer was deemed inoperable. The patient's dyspnea was alleviated by diuretics and drainage of the pleural effusion. Chemotherapy was not administered because the patient could not tolerate the side effects. She died nearly 2 months after the diagnosis.

## Discussion

This case report emphasized the value of using multiple modalities in the diagnostic work-up of PPM. Few correlative cases have been reported to date (2). For patients with massive pericardial effusion of unknown cause, a single diagnostic method alone is insufficient. In such cases, a primary cardiac tumor needs to be considered by clinicians. CEUS is a preferable imaging modality to detect pericardial masses and distinguish the essence of the tumor in this context. Hyperenhancement on imaging suggests malignancy. Other imaging modalities, including CT, CMR, and PET/CT, can be used to further assess the local extension and metastasis of the disease. Histologic examination is considered as the reference standard for establishing the final diagnosis in such cases.

Primary pericardial mesothelioma is an extremely rare malignant tumor that has a reported incidence of 0.0022%, and up to 75% of cases are diagnosed after death (3, 4). Antemortem diagnosis is uncommon because of the diverse presentations and non-specific imaging features. The prognosis of PPM is dismal, with a median survival time of 6 months from the initial symptoms (1). No satisfactory treatment strategy has been established. Patients with PPM develop diverse clinical symptoms ranging from chest pain, dyspnea, and weight loss to orthopnea, coughing, and edema. Because of the lack of sensitivity and specificity in clinical manifestations and laboratory indices, early diagnosis of PPM is notoriously difficult.

Transthoracic echocardiography is often the first choice in detecting the etiology because of its rapid use and widespread availability. In one study, pericardial effusion was the most common echocardiographic finding of PPM, followed by masses in the pericardial sac and pericardial thickening (5). Nevertheless, the detection rate of PPM using TTE is low because of the large amount of effusion, especially in the emergency bedside setting. In the present case, the small mass coexisted with a large volume of pericardial effusion and was initially overlooked. Another major limitation of TTE is its inability to distinguish between thrombi and benign or malignant tumors.

Contrast-enhanced ultrasonography can provide deeper insight into the differential diagnosis of pericardial masses. Previous studies have shown that CEUS is safe to identify, characterize and stage tumors (6). Compared with benign lesions, malignant tumors tend to show irregular shapes, an unclear boundary with the surrounding tissue, and uneven contrast enhancement, which is much greater than that of the adjacent normal myocardium (7). As in our case, PPM may be confused with massive pericardial effusion by traditional TTE. CEUS displays contrast perfusion imaging that is helpful for differentiating between malignant and benign tumors.

However, because of the limitation of the echo window, CEUS is unable to depict tumors in the mediastinum; it also fails to detect tumor invasion into the adjacent tissues. Other imaging methods are needed for further assessment.

The CMR plays a key role in the evaluation of suspected cardiac tumors. Based on excellent soft-tissue contrast and high spatial resolution, CMR can identify the anatomical localization of cardiac mass and its relationship to surrounding structures. It can also depict the tumor infiltration and the presence of fibrotic or necrotic tissue components features. According to previous studies, PPM is homogeneously isointense on T1-weighted images, heterogeneous on T2-weighted images, and gadolinium-enhanced (8–10). Furthermore, CMR has high accuracy in the exclusion of cardiac tumors (11). On the other hand, the long inversion time sequence can accurately distinguish thrombi from tumors based on the presence of a vascular supply (11). In addition, CMR is still likely to miss very small and mobile masses. Instead of a single modality “one-stop-shop” method, a multimodal imaging approach is considered to be more appropriate and accurate (12). The cardiac CT might serve as an alternative method. CT scans can demonstrate the extent of cardiac tumors, thickening of the pericardium, mediastinal lymphadenopathy, and extracardiac lesions (13).

The 18F-fluorodeoxyglucose PET/CT is an alternative tool for the diagnosis and staging of PPM. Increased FDG metabolism within the tumor may be evident prior to the appearance of anatomical changes (14). PET/CT can distinguish between malignant and benign lesions based on the diverse standardized uptake value of FDG. PET/CT is essential for detecting lymph node involvement and insidious distant metastasis, which may be normal on CT or CMR (15). Notably, the knowledge base regarding PET/CT diagnosis of PPM is limited and based mainly on a few case reports. Further investigation is needed to determine whether PET/CT can accurately stage the locoregional extent of the tumor and evaluate distant metastasis.

The final diagnosis of PPM is based on pathologic examination. Experienced pathologists and FISH are needed to achieve the diagnosis. Because the clinical presentations and laboratory abnormalities of PPM are generally non-specific, imaging methods are critically important for the early diagnosis of PPM.

## Conclusion

In this study, we reported a case in which multiple modalities were used in the diagnostic work-up of PPM. TTE is considered the first-line imaging method because of its widespread availability and rapidity, especially in the emergency bedside setting. If possible, CEUS should be applied to determine the characteristics of the mass with high accuracy based on the blood flow to increase the possibility of early identification. Moreover, further imaging, including CT, CMR, and PET/CT, may help to detect the primary tumor sites and exclude regional invasion and distant metastases.

## Data Availability Statement

The raw data supporting the conclusions of this article will be made available by the authors, without undue reservation.

## Ethics Statement

Written consent for submission and publication of this case report, including all images and associated text, in Frontiers in Cardiovascular Medicine has been obtained from the patient in line with ICMJE guidance.

## Author Contributions

JL, ZW, and BZ designed the study. YY supplied the ultrasound images. YX and WW offered reliable data for the pathologic diagnosis. JQ and KZ supplied the magnetic resonance images. JL wrote the paper. ZW and BZ revised the manuscript. All authors contributed to the article and approved the submitted version.

## Conflict of Interest

The authors declare that the research was conducted in the absence of any commercial or financial relationships that could be construed as a potential conflict of interest.

## Publisher's Note

All claims expressed in this article are solely those of the authors and do not necessarily represent those of their affiliated organizations, or those of the publisher, the editors and the reviewers. Any product that may be evaluated in this article, or claim that may be made by its manufacturer, is not guaranteed or endorsed by the publisher.
