# Supplementary material for: Multimodal Diagnostic Workup of Primary Pericardial Mesothelioma: A Case Report
Source: Front Cardiovasc Med. 2021 Nov 19;8:758988. doi: 10.3389/fcvm.2021.758988 (PMC8639695; doi:10.3389/fcvm.2021.758988)
Supplement: Supplementary file 1 [file Table_1.DOCX]

Our

hospital

**(No.1)**

**July 2020**

Dyspnea

TTE

**Oct 2020**

Worsening dyspnea

CEUS

Biopsy

Local hospital

**(No.1)**

The patient scheduled to undertake palliative chemotherapy
